# Time dependent neuroprotection of mycophenolate mofetil: effects on temporal dynamics in glial proliferation, apoptosis, and scar formation

**DOI:** 10.1186/1742-2094-9-89

**Published:** 2012-05-08

**Authors:** Fahim Ebrahimi, Marco Koch, Philipp Pieroh, Chalid Ghadban, Constance Hobusch, Ingo Bechmann, Faramarz Dehghani

**Affiliations:** 1 Institute of Anatomy, Leipzig University, 04103, Leipzig, Germany

**Keywords:** Neuroprotection, Immunosuppression, Organotypic hippocampal slice cultures, Mycophenolate mofetil, Inosine 5-monophosphate dehydrogenase, Excitotoxicity, Apoptosis, Proliferation, Scratch-wound model

## Abstract

**Background:**

Immunosuppressants such as mycophenolate mofetil (MMF) have the capacity to inhibit microglial and astrocytic activation and to reduce the extent of cell death after neuronal injury. This study was designed to determine the effective neuroprotective time frame in which MMF elicits its beneficial effects, by analyzing glial cell proliferation, migration, and apoptosis.

**Methods:**

Using organotypic hippocampal slice cultures (OHSCs), temporal dynamics of proliferation and apoptosis after N-methyl-*D*-aspartate (NMDA)-mediated excitotoxicity were analyzed by quantitative morphometry of Ki-67 or cleaved caspase-3 immunoreactive glial cells. Treatment on NMDA-lesioned OHSCs with mycophenolate mofetil (MMF)100 μg/mL was started at different time points after injury or performed within specific time frames, and the numbers of propidium iodide (PI)^+^ degenerating neurons and isolectin (I)B_4_^+^ microglial cells were determined. Pre-treatment with guanosine 100 μmol/l was performed to counteract MMF-induced effects. The effects of MMF on reactive astrocytic scar formation were investigated in the scratch-wound model of astrocyte monolayers.

**Results:**

Excitotoxic lesion induction led to significant increases in glial proliferation rates between 12 and 36 hours after injury and to increased levels of apoptotic cells between 24 and 72 hours after injury. MMF treatment significantly reduced glial proliferation rates without affecting apoptosis. Continuous MMF treatment potently reduced the extent of neuronal cell demise when started within the first 12 hours after injury. A crucial time-frame of significant neuroprotection was identified between 12 and 36 hours after injury. Pre-treatment with the neuroprotective nucleoside guanosine reversed MMF-induced antiproliferative effects on glial cells. In the scratch-wound model, gap closure was reached within 48 hours in controls, and was potently inhibited by MMF.

**Conclusions:**

Our data indicate that immunosuppression by MMF significantly attenuates the extent of neuronal cell death when administered within a crucial time frame after injury. Moreover, long-lasting immunosuppression, as required after solid-organ transplantation, does not seem to be necessary. Targeting inosine 5-monophosphate dehydrogenase, the rate-limiting enzyme of purine synthesis, is an effective strategy to modulate the temporal dynamics of proliferation and migration of microglia and astrocytes, and thus to reduce the extent of secondary neuronal damage and scar formation.

## Background

Acute pathologies of the central nervous system (CNS) such as traumatic brain and spinal cord injury, excitotoxicity, or cerebral ischemia cause immediate and irreversible damage to neurons, often associated with severe neurological impairment. All acute lesions trigger pathophysiological cascades that additionally involve delayed loss of primarily unaffected neuronal cell populations, the process known as secondary damage [1-4]. Within the scope of this phenomenon, highly complex and dynamic neuron–glia interactions lead to the activation, proliferation, and recruitment of astrocytes, microglial cells, and blood-borne immune cells. To a certain extent, inflammatory cascades carried by direct cell–cell interactions via contact-dependent communication (such as Ephrin receptors or repulsive guidance molecules) determine the mode and dimension of glial activation, glutamate sensitivity or axonal regrowth [5-8]. Furthermore, the release of a variety of soluble factors from activated glial cells modulates neuronal injury and recovery, as shown for pro- and anti-inflammatory cytokines, nitric oxide, chemokines, growth factors, prostaglandins, and reactive oxygen species [9-14]. Consequently, neuroinflammatory responses in the aftermath of acute neuropathologies may have detrimental effects, suggesting that an inhibition of glial activation by means of immunosuppression may be beneficial for neuronal survival and recovery [15-17].

Immunosuppressive drugs have been studied with regard to their capacity to arrest secondary injury cascades in a variety of models of acute CNS injury or ischemia. However, the widespread use of steroids such as methylprednisolone as acute pharmacological intervention after spinal-cord injuries has recently been questioned because of reports of only marginal effects on neurological outcome in clinical trials in opposition to the earlier promising experimental evidence [18-20]. Other immunosuppressant agents such as the immunophilin ligands ciclosporin A and FK506 (tacrolimus) likewise produced improvement neuronal survival and axonal regeneration, and have been widely investigated in animal models of acute CNS injury or ischemia [16,21-24].

The immunosuppressant mycophenolate mofetil (MMF) is clinically established for prevention of allograft rejection after solid-organ transplantation [25]. After administration, the morpholino-ester pro-drug is immediately hydrolyzed to the active compound mycophenolic acid, which in turn is a selective and reversible inhibitor of inosine 5-monophosphate dehydrogenase (IMPDH), the rate-limiting enzyme of the *de novo* purine-synthesis pathway [26-28]. The non-competitive inhibition is five-fold more potent with the type II isoform of IMPDH (IMPDH2), which is preferentially upregulated in activated leucocytes, whereas isoform I is constitutively expressed in most cell types, resulting in an almost specific inhibition of proliferation in activated leucocytes [29].

We have previously shown that MMF exerts a potent neuroprotective activity on excitotoxically lesioned organotypic hippocampal slice cultures (OHSCs) and inhibits microglial and astrocytic proliferation when administered concomitantly with lesion induction [30]. In addition, MMF effectively suppresses lipopolysaccharide (LPS)-stimulated microglial and astrocytic activation and consecutive secretion of pro-inflammatory mediators [31] by inhibition of enzymatic activity of inducible nitric oxide synthase, among other effects [32]. Furthermore, treatment with mycophenolate mofetil strongly improves the preservation of myelinated long-range projections *in vitro*[33] and axonal regeneration *in vivo*[34].

These promising studies raise the possibility that immediate MMF administration might be useful for the treatment of acute CNS lesions. However, little is known about the effective time-frame in which MMF treatment can still be applied without inducing widespread immunosuppression.

Thus, we assessed the effects of MMF on the temporal dynamics of microglial and astrocytic proliferation and apoptosis after acute excitotoxicity in OHSCs, and analyzed MMF-mediated effects on scar formation in the scratch-wound model.

## Methods

All animal experiments were performed in accordance with the Policy on Ethics and the Policy on the Use of Animals in Neuroscience Research as approved by the directive 2010/63/EU of the European Parliament and of the Council of the European Union on the protection of animals used for scientific purposes.

### Preparation and maintenance of organotypic hippocampal slice cultures

For the preparation of OHSCs, 8-day-old Sprague–Dawley rats were decapitated, and the brains were dissected under sterile conditions according to standard protocols [35]. After removal of the frontal pole and the cerebellum, the brains were placed in preparation medium at 4°C; this preparation medium consisted of minimal essential medium (MEM; Gibco BRL Life Technologies, Eggenstein, Germany) pH 7.35 including 1% v/v glutamine (Gibco). Subsequently, these preparations were sliced into sections 350 μm thick on a vibratome (Vibratom VT 1200 S; Leica Microsystems AG, Wetzlar, Germany). Approximately six to eight OHSCs were obtained from each brain, and were immediately transferred into cell- culture inserts with a pore size of 0.4 μm (Falcon, BD Bioscience Discovery Labware, Bedford, MA, USA), which were placed in six-well culture dishes (Falcon) and fed with 1 ml culture medium per well. The culture medium consisted of 50% v/v MEM, 25% v/v Hanks’ balanced salt solution supplemented with 185 mg/L CaCl_2_ and 100 mg/L MgCl_2_, 25% v/v normal horse serum (all Gibco), 2% v/v glutamine, 1 μg/ml insulin (Boehringer, Mannheim, Germany), 1.2 mg/ml glucose (Braun, Melsungen, Germany), 0.1 mg/ml streptomycin, 100 U/ml penicillin and 0.8 μg/ml vitamin C (Sigma-Aldrich Chemicals, Deisenhofen, Germany) at pH 7.4. The culture dishes were incubated for 6 days at 35°C in a fully humidified atmosphere with 5% CO_2_ and the culture medium was changed on alternate days. Mycophenolate mofetil (CellCept; Roche, Grenzach-Wyhlen, Germany) was diluted in cell-culture medium for application to OHSCs at a previously determined effective concentration of 100 μg/ml [30]. The nucleoside guanosine (Sigma-Aldrich Chemicals) was applied at a concentration of 100 μmol/l at 6 days *in vitro* before MMF treatment to counteract MMF-induced purine depletion.

### Treatment protocols of organotypic hippocampal slice cultures

The preparations were randomly divided into different experimental groups and treated according to the following protocols:

#### Control

Unlesioned OHSCs (n = 57) served as control slices, and were kept in culture medium for 9 days *in vitro* without any treatment.

#### NMDA

At 6 days *in vitro*, OHSCs (n = 43) were lesioned with NMDA (50 μmol/l; Sigma-Aldrich Chemicals) for 4 hours, and thereafter kept in culture medium for another 3 days *in vitro*.

#### NMDA time series

**Figure 1:**
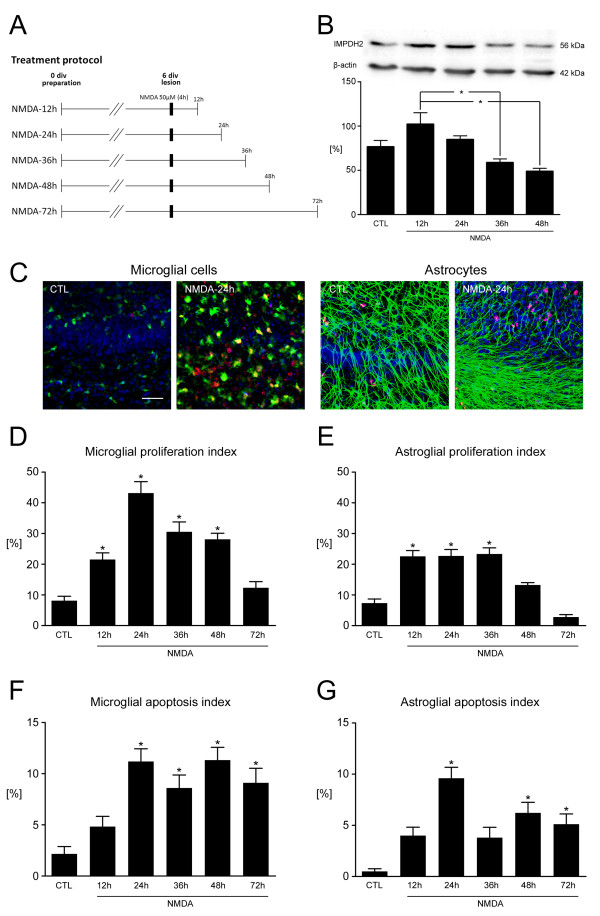
**Temporal dynamics of cellular responses after excitotoxic lesion. (A)** Treatment protocols. **(B)** (Upper panel) Immunoblot analyses of inosine 5-monophosphate dehydrogenase (IMPDH)2 and β-actin at different time points post-lesion. *N*-methyl-D-aspartate (NMDA) lesion affected IMPDH2 immunoreactivity over time. (Lower panel) Semiquantitative analyses of immunoblot data (n = 4) showed a significant reduction in the amount of IMPDH2 at 36 and 48 hours after injury compared with the values at 12 hours (*P < 0.05). **(C)** Confocal laser scanning microscopy images, double-labeled with isolectin (I)B_4_ (microglial cells, green) or glial fibrillary acidic protein (GFAP) (astrocytes, green) each in combination with Ki-67 (proliferating cells, red). Temporal patterns of **(D)** microglial and **(E)** astroglial proliferation indices after NMDA-mediated excitotoxic lesion as shown by quantitative morphometry of Ki-67^+^ glial cell ratios (*P < 0.05 vs. control (CTL)). Microglial proliferation indices were significantly increased from 12 hours to 48 hours, with maximum values at 24 hours post-lesion. Astrocytic proliferation indices were significantly increased from 12 hours to 36 hours after injury. **(F–G)** Microglial and astroglial apoptosis indices after NMDA-mediated excitotoxic lesion as measured by assessment of ratios of cleaved caspase-3^-^immunoreactive glial cells (*P < 0.05 vs. control (CTL)). The apoptosis indices of both microglial cells and astrocytes were significantly increased between 24 and 72 hours, with a decline at 36 hours post-lesion. Scale bar = 50 μm

At 6 days *in vitro*, OHSCs were lesioned with NMDA for 4 hours, and subsequently fixed at 12, 24, 36, 48 or 72 hours after lesion induction (Figure 1 A).

#### Continuous MMF treatment after injury

**Figure 2:**
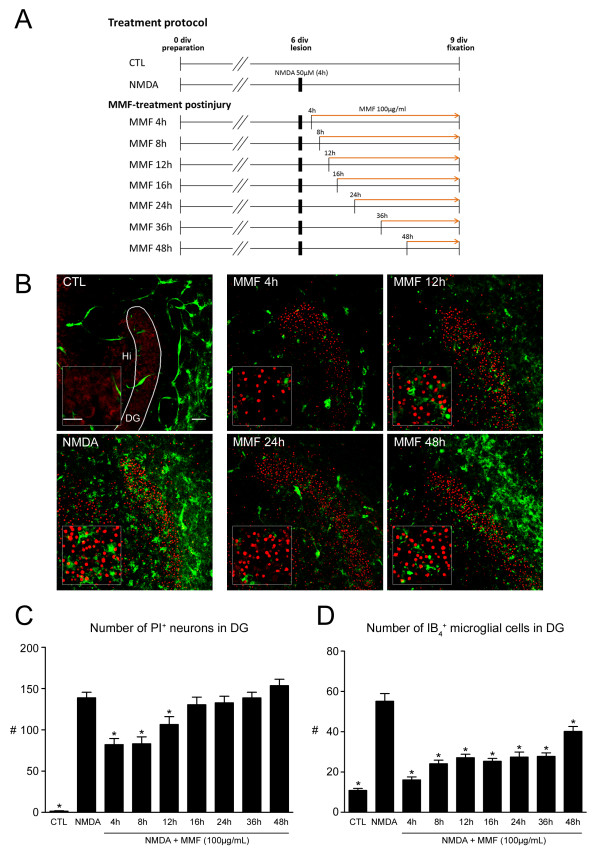
**Effects of continuous mycophenolate mofetil (MMF) treatment from different time points after injury on neuronal survival and microglial activation in organotypic hippocampal slice cultures (OHSCs).(A)** Treatment protocols. **(B)** Confocal laser scanning microscopy images in overview and higher magnification, double-labeled with propidium iodide (PI) (degenerating neurons, red) and IB_4_ (microglial cells, green). Untreated control (CTL) OHSCs had very good preservation of the hippocampal formation with almost no PI^+^ pyknotic nuclei and only a few ramified IB_4_^+^ microglial cells. Slices treated with NMDA 50 μmol/l for 4 hours had massive accumulation of amoeboid IB_4_^+^ microglial cells and a dramatic increase in PI^+^ neuronal nuclei. Delayed treatment (until 12 hours after injury) with MMF resulted in a significant reduction of PI^+^ degenerating neurons. Numbers of microglial cells were reduced even with late administration of MMF (after 48 hours post-injury). **(C-D)** Quantitative analyses of the effects of MMF on NMDA-lesioned OHSCs. The mean numbers of **(C)** PI^+^ degenerating neurons and **(D)** IB_4_^+^ microglial cells were calculated for each experimental group and compared with OHSCs treated with NMDA alone (*P < 0.05 vs. NMDA). DG, dentate gyrus; Hi, hilus. Scale bars = 50 μm in overviews; 20 μm in insets

OHSCs were kept in culture medium for 6 days *in vitro*, lesioned with NMDA (50 μmol/l) for 4 hours, and treated with MMF 100 μg/ml at different starting time points after injury (4 hours, n = 17; 8 hours, n = 20; 12 hours, n = 24; 16 hours, n = 15; 24 hours, n = 18; 36 hours, n = 16; 48 hours, n = 17) for another 3 days *in vitro*, (Figure 2A).

#### Time frame of MMF treatment after injury

**Figure 3:**
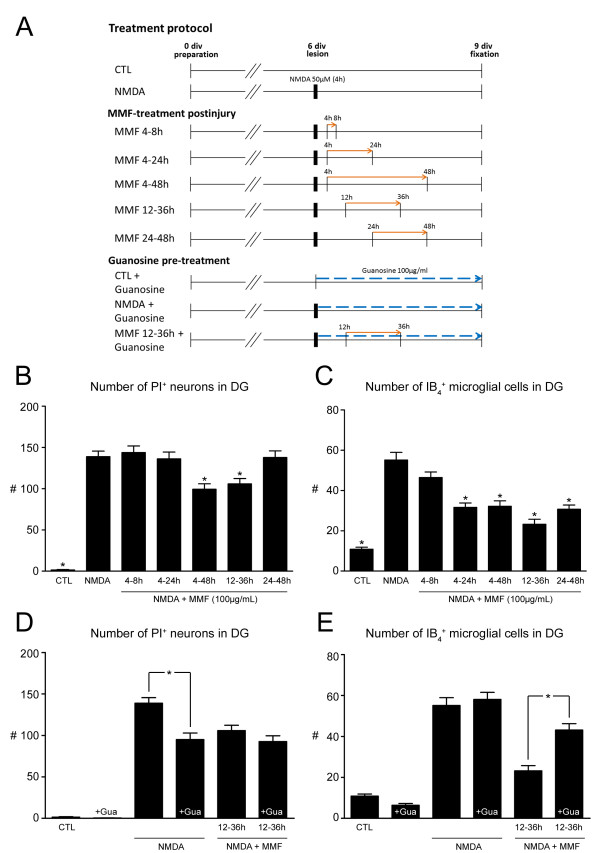
**Mycophenolate mofetil (MMF) treatment in specific time frames after injury and effects of guanosine on neuroprotective and antiproliferative properties of MMF.(A)** Treatment protocols. **(B,C)** Quantitative analyses of the effects of short-term MMF treatment (100 μg/ml) on neuronal survival and microglial activation in organotypic hippocampal slice cultures (OHSCs) when MMF was administered in different time windows after injury. Mean numbers of **(B)** propidium iodide (PI)^+^ degenerating neurons and **(C)** IB_4_^+^ microglial cells were calculated for each experimental group, and compared with OHSCs treated with *N*-methyl-D-aspartate (NMDA) alone (*P < 0.05 vs. NMDA). MMF elicits strong neuroprotective effects when administered within the time frame of 4 to 48 hours or even 12 to 36 hours after injury. However, treatment within 4 to 24 hours or 24 to 48 hours after injury likewise reduced numbers of IB_4_^+^ microglial cells. **(D,E)** Quantitative analyses of guanosine effects. Mean numbers of **(D)** PI^+^ degenerating neurons and **(E)** IB_4_^+^ microglial cells were calculated for each experimental group and compared with matched OHSCs that did not receive guanosine (*P < 0.05). Guanosine did not affect either neuronal viability or microglial activation when applied to unlesioned control (CTL) OHSCs. In NMDA-lesioned OHSCs subjected to guanosine treatment, there was a significant reduction in the numbers of PI^+^ degenerating neurons, whereas the numbers of IB_4_^+^ microglial cells were unaffected. However, application of guanosine to OHSCs that received MMF treatment within the 12 to 36 hour time window significantly reversed the antiproliferative reduction in IB_4_^+^ microglial cells without affecting MMF-dependent neuroprotection

OHSCs were kept in culture medium for 6 days *in vitro*, lesioned with NMDA (50 μmol/l) for 4 hours and thereafter treated with MMF (100 μg/ml) within a certain time window (i) 4 to 8 hours after injury (n = 21), (ii) 4 to 24 hours after injury (n = 26), (iii) 4 to 48 hours after injury (n = 15), (iv) 12–36 hours after injury (n = 23) and (v) 24 to 48 hours after injury (n = 15), (Figure 3A).

### Determination of neuronal cell death and confocal laser scanning microscopy

To visualize the pyknotic nuclei of degenerating neurons, OHSCs at 9 days *in vitro* were incubated with PI 5 μg/ml (Chemicon, Nuernberg, Germany) for 2 hours before fixation. The use of PI as an indicator for cell viability and for identification of degenerating neurons in OHSCs has been established previously [36,37]. After rinsing with 0.1 mol/l phosphate buffer, slices were fixed with a 4% w/v solution of paraformaldehyde in 0.2 mol/l phosphate buffer overnight.

The pre-fixed OHSCs were then removed from the cell culture inserts, placed into 24-well plates (Falcon), and washed for 10 minutes with PBS containing 0.03% v/v Triton X-100 (PBS/Triton X-100) for 10 minute. The slices were then incubated with normal goat serum (diluted 1:20 in PBS/Triton X-100) for 1 hours, and stained with fluorescein isothiocyanate (FITC)-conjugated *Griffonia simplicifolia* IB_4_ (Vector laboratories, Burlingame, CA, USA) diluted 1:50 in PBS/Triton X-100 containing 0.25% (w/v) bovine serum albumin for 3 hours. The slices were washed with PBS/Triton X-100 for 10 minute and finally mounted under coverslips using fluorescent mounting medium (Dako Diagnostika GmbH, Hamburg, Germany).

OHSCs were analyzed and imaged with a confocal laser scanning microscope (LSM 510 Meta, Zeiss, Goettingen, Germany). For detection of PI^+^ nuclei of degenerating neurons, monochromatic light at 543 nm and an emission bandpass filter of 585 to 615 nm were used. For visualization of IB_4_^+^ microglial cells, monochromatic light at 488 nm with a dichroic beam splitter (FT 488/543) and an emission band-pass filter of 505 to 530 nm were used.

Confocal images were obtained at 160-fold magnification at a resolution of 1024 × 1024 pixels. Using the mid-stag mode and the Z-mode of the confocal microscope, the optical mid-stag and the two adjacent optical sections (2 μm thick) of the granule cell layer (GCL) in the dentate gyrus (DG) were obtained and converted into a binary image. Subsequently, numbers of IB_4_^+^ microglial cells and PI^+^ degenerating neurons were counted in the GCL of the DG (cells/GCL) as previously described [14,37,38]. Lesioned OHSCs treated with MMF at different time points or within specific time frames after injury were compared with OHSCs treated with NMDA alone.

### Analyses of microglial and astrocytic proliferation and apoptosis

For analyses of proliferation and apoptosis indices of microglial cells and astrocytes, NMDA-lesioned OHSCs were fixed at 12, 24, 36, 48 and 72 hours after injury.

Quadruple staining was performed using GFAP, IB_4_, 4’-6-diamidino-2-phenlyindole (DAPI) and Ki-67 or cleaved caspase-3, respectively. OHSCs were removed from the cell-culture insert membranes, washed with PBS, and cryoprotected with ascending solutions of 10%, 20% and 30% w/v sucrose before being sectioned horizontally at 12 μm thickness on a cryostat (CM3050 S; Leica) at −23°C. Obtained sections were subsequently mounted on microscope slides (Superfrost Plus; Gerhard Menzel GmbH, Braunschweig, Germany) and air-dried. Cryostat sections were washed with PBS/Triton X-100 for 10 minutes, pre-incubated with normal goat serum (diluted 1:20 in PBS/Triton) for 30 minutes and incubated with the primary antibody (rabbit anti-Ki-67, diluted 1:200; DCS Innovative Diagnostik Systeme, Hamburg, Germany) or (rabbit anti-cleaved caspase-3, diluted 1:200; Cell Signalling Technology, Danvers, MA, USA) for 12 hours. After washing with PBS-Triton X-100, the slices were simultaneously incubated with the secondary antibody (1:200, Alexa 568-conjugated goat anti-rabbit IgG; Invitrogen) for 1 hour. Sections were then incubated with a monoclonal rat anti-GFAP antibody (1:200, Dako) for 12 hours, washed, and incubated with the secondary goat anti-rat Alexa 633-conjugated antibody (1:200, Invitrogen) for 1 hour. Microglial cells were stained with FITC-IB_4_ for 1 hour as described above. Finally, nuclear staining was performed by application of DAPI 100 ng/mL (Molecular Probes, Mobitec, Goettingen, Germany) for 15 minutes.

Sections were then washed with PBS-Triton and distilled water for 10 minutes each, coverslipped with fluorescent mounting medium (Dako) and analyzed by confocal laser scanning microscopy in multi-tracking mode. Monochromatic light at 488, 543, and 633 nm with a dichroic beam splitter (FT UV/488/543/633) was used to visualize cellular nuclei (DAPI; excitation 405 nm, emission band-pass filter 420 to 480 nm), microglial cells (FITC-IB_4_; excitation 488 nm, emission band-pass filter 505 to 530 nm), astrocytes (GFAP; excitation 633 nm, emission long pass 650 nm), proliferating cells (Ki-67; excitation 543 nm, emission band-pass filter 585 to 615 nm) or cleaved caspase-3^+^ immunoreactive cells (cleaved caspase-3; excitation 543 nm, emission band-pass filter 585 to 615 nm).

### Quantitative morphometry of organotypic hippocampal slice cultures

Quantitative morphometry was used for determination of the following parameters: 1) the number of PI^+^ degenerating neurons, 2) the number of IB_4_^+^ microglial cells, 3) the number of Ki-67^+^ proliferating cells and 4) the numbers of cleaved caspase-3^+^ immunopositive cells each throughout the entire granule-cell layer of the DG using ImageJ software (ImageJ 1.44p, National Institutes of Health, USA). Proliferation indices were calculated as the percentage of Ki-67^+^ proliferating microglial cells or astrocytes related to the total number of respective glial cells in DG. Correspondingly, apoptosis indices were determined as the percentage of cleaved caspase-3^+^ immunopositive glial cells per total numbers of glial cells.

### Immunoblotting and semiquantitative analyses

For immunochemical analyses, hippocampal formations of differentially treated OHSCs were used. Protein concentrations were determined by the dye-based method of Bradford [39], and equivalent quantities were loaded onto 12.5% SDS–PAGE gels. After gel electrophoresis, the proteins were blotted onto nitrocellulose membranes, which were then pre-incubated in blocking buffer (5% milk, 25 mmol/l Tris–HCl, 150 mmol/l NaCl) pH 7.5 to reduce non-specific binding of the antibody. The nitrocellulose membranes were incubated overnight with the aforementioned antibody against IMPDH2 (diluted 1:2000 in blocking buffer containing 0.2% Tween 20). Binding of the primary antibody was visualized using horseradish peroxidase-conjugated goat anti-rabbit IgG and enhanced chemiluminescence (Thermo Fisher Scientific, Barrington, IL, USA). For semiquantitative analysis, the relative signal intensities of the immunoreactive bands were determined and standardized as quotients of IMPDH2 intensity units per β-actin intensity units.

### Primary astrocyte cell cultures and scratch-wound model analyses

Astrocyte cell cultures were prepared from p0-2 Sprague–Dawley rat brains according to standard protocols [40]. In brief, brains were treated with trypsin (4 mg/mL; Boehringer) in Hank’s balanced salt solution without Ca^2+^ or Mg^2+^ with DNAse (0.5 mg/mL; Worthington, Bedford, MA, USA), and resuspended in 1 mL culture medium, consisting of DMEM (Gibco), supplemented with 10% FCS (Gibco), 1% glutamine (Boehringer), 100 U/mL penicillin and 0.1 mg/mL streptomycin (Sigma-Aldrich Chemicals). The cell suspension was transferred into tissue culture flasks (75 cm^2^; Falcon) and incubated for 7 days *in vitro*. Astrocytic monolayers were subsequently treated with trypsin in PBS without Ca^2+^ or Mg^2+^, and seeded onto poly-*L*-lysine coated coverslips in 24-well tissue culture plates (Falcon). Purity of astrocytic cultures was verified by immunocytochemistry against GFAP (GFAP^+^ astrocytes > 98%).

For *in vitro* scratch-wound model experiments, confluent astrocyte monolayers were wounded by scratching with sterile plastic pipette tips. Scratched cultures remained either untreated (control) or were treated with MMF at a concentration of 1 μg/mL. At 0, 2, 6, 12, 24 and 48 hours after scratching, astrocytes were fixed for assays on cell proliferation, using anti-GFAP and anti-Ki-67 immunofluorescence staining as described above. Proliferation indices were calculated as the percentage of Ki-67^+^ proliferating astrocytes related to the total number of astrocytes around the scratch-wound area. The mean astrocytic gap widths were measured by image analysis. The mean gap width of untreated (control) cultures at baseline (immediately after scratching; 0 hours) was defined as 100%. Data from other time points and MMF treatment were presented as percentages of the controls at baseline.

### Statistical analysis

Data are presented as mean ± SEM. For statistical analysis the one-way ANOVA test was used followed by Dunnett’s *post hoc* test or Bonferoni’s test for multiple comparisons when the effect of MMF treatment on numbers of PI^+^ degenerating neurons, IB_4_^+^ microglial cells, Ki-67^+^ proliferating cells, cleaved caspase-3-positive cells, IMPDH signal intensities or gap widths, respectively, was significant. *P* < 0.05 was considered significant.

## Results

### Temporal dynamics of proliferation and apoptosis after acute excitotoxic injury

To investigate temporal patterns of glial cell proliferation and apoptosis, indices of Ki-67 and cleaved caspase-3 immunoreactive astrocytes and microglial cells were assessed at different proximate time points after the onset of acute excitotoxic lesion.

Unlesioned control OHSCs displayed low levels of Ki-67^+^ proliferating microglial cells or astrocytes (8.1% and 7.3%, respectively) (Figure 1D,E). Correspondingly, apoptosis indices of control OHSCs were markedly low: only 2.2% microglial cells and 0.5% astrocytes were immunoreactive for cleaved caspase-3 (Figure 1F,G). After NMDA-mediated excitotoxic lesion induction of OHSCs, the fraction of proliferating microglial cells rapidly increased within the first 12 hours post-lesion, and the proliferation index reached its maximum at 24 hours after injury (12 hours: 21.5%, *P* < 0.01; 24 hours: 43.2%, *P* < 0.0001) (Figure 1 D). Thereafter the fraction of proliferating microglial cells decreased from 31.7% at 36 hours to 28.1% at 48 hours after injury (*P* < 0.001) and reached almost control levels at 72 hours after injury (11.2% Ki-67^+^ microglial cells, *P* > 0.05) (Figure 1D). Similarly, astroglial proliferation indices increased most rapidly within the first 12 hours post-lesion and reached a plateau up to 36 hours (12 hours: 22.8%; 24 hours: 22.7%; 36 hours: 23.3%, *P* < 0.001), and thereafter fractions of proliferating astrocytes steadily declined (48 hours: 13.3%; 72 hours: 2.8%, *P* >0.05) (Figure 1E).

In the aftermath of NMDA-induced excitotoxic injury, the ratios of glial cells undergoing apoptotic conditions increased steadily over time. At 12 hours post-lesion, the microglial apoptosis indices remained unaltered compared with unlesioned control OHSCs, but the ratio significantly increased within 24 hours and continued to increase until 72 hours after the initial lesion (12 hours: 4.8%, *P* > 0.05; 24 hours: 11.2%, *P* < 0.0001; 36 hours: 8.6%, *P* < 0.01; 48 hours: 11.3%, *P* < 0.0001; 72 hours: 9.1%, *P* < 0.001) (Figure 1F). The apoptosis indices of astrocytes were similar to those seen with microglial cells, but with significantly diminished apoptosis ratio at 36 hours post-lesion (12 hours: 4.0%, *P* > 0.05; 24 hours: 9.6%, *P* < 0.0001; 36 hours: 3.8%, *P* > 0.05; 48 hours: 6.2%, *P* < 0.001; 72 hours: 5.1%, *P* < 0.01) (Figure 1G).

### Inosine 5-monophosphate dehydrogenase 2 immunoreactivity after *N*-methyl-D-aspartate-induced lesion and mycophenolate mofetil treatment

IMPDH2 immunoreactivity was assessed in immunoblots at different time points after NMDA-mediated lesion induction of OHSCs at 6 days *in vitro*. The antibody against IMPDH2 labeled a single band of 56 kDa, corresponding to the known molecular weight of the enzyme. This band was found in unlesioned control OHSCs and its intensity gradually changed after NMDA application when assessed at 12, 24, 36 and 48 hours post-lesion. IMPDH2 signal intensity standardized to β-actin was significantly increased at 12 hours post-lesion compared with intensities at 36 and 48 hours after injury (*P* < 0.05) (Figure 1B).

### Continuous mycophenolate mofetil treatment of *N*-methyl-D-aspartate-lesioned organotypic hippocampal slice cultures from different time points

In control OHSCs, the typical hippocampal cytoarchitecture was well preserved (Figure 2B). Throughout all optical sections almost no pyknotic PI^+^-nuclei and only a few IB_4_^+^ microglial cells were found (control: 1.5 PI^+^ and 10.8 IB_4_^+^ cells/GCL) (Figure 2C,D) confirming excellent preservation after 9 days *in vitro* under standard incubation conditions. Microglial cells, which were primarily found in the molecular and plexiform layers of the DG and hippocampus, exhibited a ramified phenotype with highly branched processes.

OHSCs treated after 6 days *in vitro* for 4 hours with 50 μmol/l NMDA and analyzed after 9 days *in vitro* showed massive neuronal damage, reflected by greatly increased numbers of PI^+^ degenerating neurons exhibiting luminously stained condensed nuclei with occasional signs of karyorrhexis in the GCL of the DG (Figure 2B). Quantitative analysis of IB_4_^+^ microglial cells confirmed that the number of microglial cells was likewise significantly increased compared with controls (NMDA: 138.9 PI^+^ and 55.1 IB_4_^+^ cells/GCL, *P* < 0.001) (Figure 2C,D).

Continuous treatment of lesioned OHSCs with MMF 100 μg/ml significantly reduced the numbers of both PI^+^ degenerating neurons and of IB_4_^+^ microglial cells in a time-dependent manner (Figure 2C,D). When MMF treatment was started at 4 hours after injury, the number of PI^+^ pyknotic nuclei of degenerating neurons was significantly decreased (4 hours: 82.1/GCL, *P* < 0.001) (Figure 2B,C) compared with NMDA-lesioned cultures only (NMDA: 138.9/GCL). The mean number of IB_4_^+^ microglial cells correspondingly decreased significantly, dropping from 55.1 cells/GCL to 16.1 cells/GCL (*P* < 0.001) (Figure 2D). Similar neuroprotective effects of MMF were seen when treatment was implemented at 8 hours after injury, which were paralleled by a significant reduction in the number of microglial cells (8 hours: 83.2 PI^+^ cells/GCL, 24.1 IB_4_^+^ cells/GCL, *P* < 0.001) (Figure 2C,D). MMF administration from 12 hours post-lesion still resulted in significant neuroprotection (12 hours: 106.5 PI^+^ cells/GCL, *P* < 0.01) (Figure 2C), but the reduction in the mean number of PI^+^ cells was less pronounced compared with the experimental groups that received MMF earlier. Application of MMF at 12 hours after injury caused a similar reduction in numbers of microglial cells compared with an application after 4 or 8 hours (12 hours: 27.1 IB_4_^+^ cells/GCL, *P* < 0.001) (Figure 2D). However, when MMF treatment was delayed until 16 hours after excitotoxic injury, the neuroprotective effects were no longer visible, and teh numbers of PI^+^ degenerating neurons in the GCL of DG (16 hours: 125.6 PI^+^ cells/GCL, *P* > 0.05) (Figure 2C) were now equivalent to those in OHSCs treated with NMDA alone, whereas the number of microglial cells was still considerably decreased (16 hours: 25.3 IB_4_^+^ cells/GCL, *P* < 0.001). Although no neuroprotective efficacy was detected in excitotoxically lesioned OHSCs given MMF at 24 (128.0 PI^+^ cells/GCL), 36 (138.8 PI^+^ cells/GCL) or 48 hours (153.6 PI^+^ cells/GCL, *P* > 0.05) (Figure 2C) after injury, statistical analysis showed that the numbers of IB_4_^+^ microglial cells were consistently reduced (24 hours: 27.4 IB_4_^+^ cells/GCL; 36 hours: 27.7 IB_4_^+^ cells/GCL; 48 hours: 40.2 IB_4_^+^ cells/GCL, *P* < 0.001) (Figure 2D) even after late initiation of MMF administration.

### Mycophenolate mofetil treatment in definite time windows after injury

Treatment of OHSCs with MMF 100 μg/ml was performed in specific time-frames to determine the window of opportunity to achieve highest neuroprotective efficacy. When MMF treatment was scheduled in an early and short time window (4 to 8 hours after injury), there was no effect on either the number of PI^+^ degenerating neurons or the number of IB_4_^+^ microglial cells in the DG compared with NMDA-lesioned OHSCs (4 to 8 hours: 143.8 PI^+^ and 46.5 IB_4_^+^ cells/GCL, *P* > 0.05) (Figure 3B,C). Administration of MMF during the window of 4 to 24 hours after injury also did not elicit neuroprotective effects on OHSCs (136.2 PI^+^ cells/GCL, *P* > 0.05) (Figure 3B), but significantly decreased the numbers of IB_4_^+^ microglial cells (31.6 IB_4_^+^ cells/GCL, *P* < 0.001) (Figure 3C).

However, application of MMF within a broader time window (4 to 48 hours after injury) significantly reduced the numbers of PI^+^ neurons and IB_4_^+^ microglial cells (4 to 48 hours: 99.3 PI^+^ and 31.5 IB_4_^+^ cells/GCL, *P* < 0.001) (Figure 3B,C). Correspondingly, a delayed and shortened MMF treatment (12 to 36 hours after NMDA lesion) significantly reduced the numbers of PI^+^ degenerating neurons and IB_4_^+^ microglial cells (12–36 hours: 105.8 PI^+^ and 23.2 IB_4_^+^ cells/GCL, *P* < 0.01) (Figure 3B,C). In subsequent experiments, this 12 to 36-hour time frame served as a positive control for highest MMF efficacy.

When MMF application was delayed to 24 hours after injury and lasted until 48 hours, the neuroprotective effects were lost, although administration within this late time window evenly reduced the mean numbers of microglial cells counted 72 hours after injury (137.8 PI^+^ and 30.7 IB_4_^+^ cells/GCL, *P* < 0.001) (Figure 3B,C).

### Antagonization of mycophenolate mofetil-induced purine depletion by pre-treatment with the nucleoside guanosine

Application of guanosine 100 μmol/l on excitotoxically lesioned OHSCs that were subjected to MMF treatment was performed to investigate a possible antagonization of the MMF-induced neuroprotective and antiproliferative effects by refilling the previously depleted purine pools in glial cells.

Guanosine treatment on unlesioned control OHSCs did not affect neuronal survival or microglial cell numbers (control ^+^ guanosine: 0.4 PI^+^ cells/GCL, 6.4 IB_4_^+^ cells/GCL, *P* > 0.05) (Figure 3D,E), but it significantly reduced the degree of neuronal cell death when applied to NMDA-lesioned OHSCs (NMDA + guanosine: 97.4 PI^+^ cells/GCL, *P* < 0.001) (Figure 3D) without affecting the number of IB_4_^+^ microglial cells (57.2 IB_4_^+^ cells/GCL, *P* > 0.05) (Figure 3E). When OHSCs subjected to MMF administration within the crucial neuroprotective 12 to 36-hour time window received guanosine pre-treatment, no changes in the neuroprotective efficacy of MMF were seen (MMF 12 to 36 hours + guanosine: 92.6 PI^+^ cells/GCL, *P* > 0.05) (Figure 3D). Similarly, there was no significant difference in the number of PI^+^ degenerating neurons compared with lesioned OHSCs treated with guanosine alone (*P* > 0.05) (Figure 3D). However, pre-treatment with guanosine potently counteracted MMF-mediated reductions in the number of microglial cells, as shown by significant increases in the number of IB_4_^+^ microglial cells in GCL of DG (MMF 12 to 36 hours + guanosine: 43.1 IB_4_^+^ cells/GCL, *P* < 0.001) (Figure 3E).

### Effects of mycophenolate mofetil on glial proliferation and apoptosis

**Figure 4:**
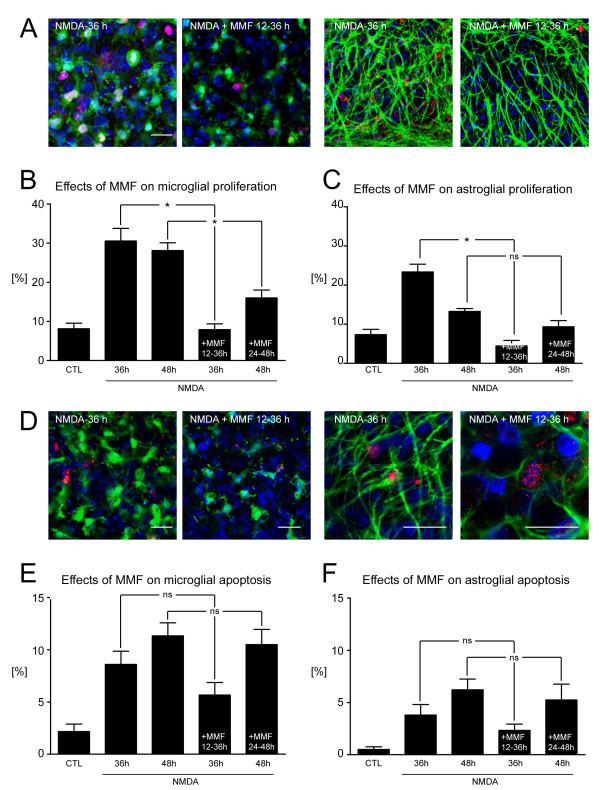
**Effects of mycophenolate mofetil (MMF) treatment on glial apoptosis and proliferation.(A)** High-magnification confocal laser scanning microscopy images, double-labeled with Ki-67 (proliferating cells, red) in combination with either isolectin (I)B_4_ (microglial cells, green) or glial fibrillary acidic protein (astrocytes, green). Effects of MMF on **(B)** microglial and **(C)** astroglial proliferation indices at 36 hours or 48 hours post-lesion compared with lesioned organotypic hippocampal slice cultures (OHSCs) that received additional MMF treatment within 12 to 36 hours or 24 to 48 hours, respectively (*P < 0.05). (**D**) Confocal laser scanning microscopy images at high magnification, double-labeled with cleaved caspase-3 (apoptotic cells, red) with either IB_4_ (microglial cells, green) or GFAP (astrocytes, green). **(E,F)** Effects of MMF on microglial and astroglial apoptosis indices at 36 or 48 hours post-lesion compared with lesioned OHSCs that received additional MMF treatment within 12–36 hours or 24 to 48 hours, respectively (*P < 0.05). MMF treatment within the crucial neuroprotective 12–36-hour time window potently attenuated microglial and astrocytic proliferation, but did not affect rates of glial apoptosis. Application of MMF within 24 to 48 hours post-lesion significantly reduced microglial proliferation but did not affect either astrocytic proliferation or glial apoptosis. Scale bars = 25 μm

MMF treatment on NMDA-lesioned OHSCs within the crucial neuroprotective 12 to 36-hour time window significantly reduced both microglial and astroglial proliferation indices assessed at 36 hours post-lesion (MMF 12 to 36 hours: 7.9% Ki-67^+^ microglial cells and 4.4% astrocytes, *P* < 0.001) (Figure 4B,C). However, although MMF administration during the later time window of 24 to 48 hours after injury also significantly reduced the fraction of Ki-67^+^ proliferating microglial cells, it did not alter the indices of astroglial proliferation assessed at 48 hours post-lesion (MMF 24 to 48 hours: 16.1% Ki-67^+^ microglial cells, *P* < 0.01; 9.3% astrocytes, *P* > 0.05) (Figure 4B,C).

Notably, treatment with MMF did not affect indices of either microglial or astroglial apoptosis when applied within the 12 to 36-hours or 24 to 48-hour time frames (MMF 12 to 36 hours: 5.7% caspase-3^+^ microglial cells and 2.3% astrocytes; MMF 24 to 48 hours: 10.5% caspase-3^+^ microglial cells and 5.3% astrocytes, *P* > 0.05) (Figure 4E,F).

### Scratch-wound model: Effects of mycophenolate mofetil on astrocyte migration and proliferation

To address the question of whether MMF can directly modulate astrogliosis, the *in vitro* scratch-wound model was used to determine the effects of MMF on mechanical injury-induced astrocyte proliferation. Ratios of Ki-67^+^ reactive astrocytes around the wounded area were evaluated to determine scratch injury-induced astrocyte proliferation.

**Figure 5:**
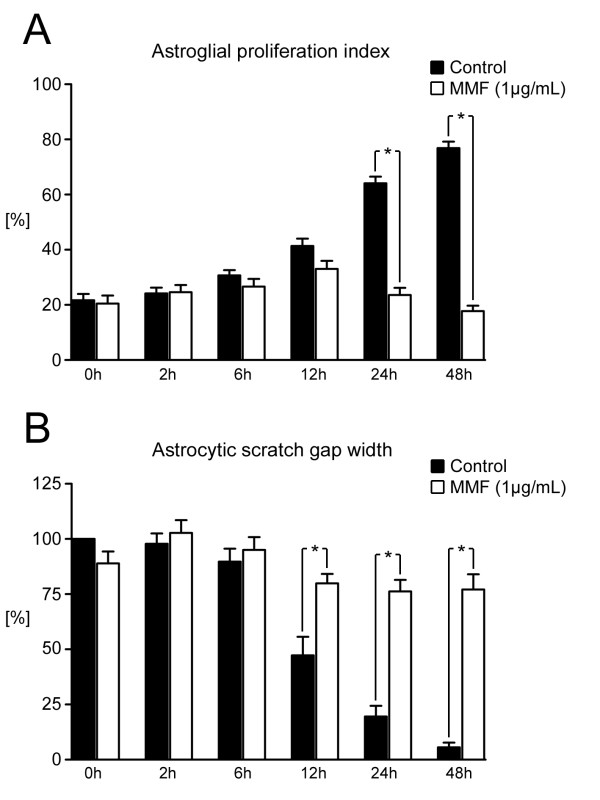
**Effects of mycophenolate mofetil (MMF) on astroglial proliferation and scar formation in*in vitro*scratch-wound model.(A)** Astroglial proliferation indices in astrocyte cell cultures after mechanical scratch injury as shown by quantitative morphometry of Ki-67^+^ astroglial cell ratios at different time points post-lesion. Untreated control cultures had significantly increased rates of proliferating astrocytes from 12 hours post-lesion compared with baseline (0 hours). Continuous treatment with MMF 1 μg/ml potently attenuated astrocytic proliferation at 24 and 48 hours post- scratching compared with matched control cultures (*P < 0.05). **(B)** Quantitative measurements of scratch-gap widths as measured by image analysis, normalized on the mean gap width of control cultures at 0 hours. Untreated control cultures had constant migration of astrocytes towards the scratch-induced gap over time, leading to gap closure at 48 hours post-lesion. MMF treatment inhibited reactive astrogliosis. No significant changes were found in gap widths compared with controls at 0 hours (*P < 0.05)

At 12 hours after scratching, the numbers of astrocytes within and next to the wound site had increased and the respective cytoplasmic processes extended to the denuded area. Cell proliferation adjacent to the wound site was significantly enhanced from 12 hours after the scratch procedure (12 hours: 41.3%, 24 hours: 64.1%, and 48 hours: 76.9% Ki-67^+^ proliferating astrocytes, *P* < 0.01) (Figure 5A). Accordingly, high rates of proliferation led to a steady closing of the gap until cell density approached confluence at 48 hours post- scratching compared with control cultures at 0 hours (*P* < 0.001) (Figure 5B). Pre-treatment of astrocytes with MMF 1 μg/ml significantly decreased the scratch injury-induced fraction of Ki-67^+^ astrocytes at 24 and 48 hours compared with controls at the same time points (24 hours: 23.6% and 48 hours: 17.8%, respectively, *P* < 0.001) (Figure 5A). The reduced rates of proliferation after MMF treatment diminished the capacity of cell migration and glial scar formation as shown by the lack of gap closure (*P* < 0.001) (Figure 5B).

## Discussion

In the aftermath of acute brain and spinal cord injuries, neuroinflammatory processes induce various cellular and molecular reactions, accompanied by rapid activation of both microglial cells and astrocytes. However, the mode and dimension of glial activation can even exacerbate the original extent of neuronal damage, with potentially harmful effects on neuronal survival, axonal regrowth, or restitution of complex network functions [41-43].

Based on this hypothesis, effective suppression of glial activation in a crucial time frame during the acute phase of pathophysiological responses would promote neuronal integrity. Several immunosuppressive substances possess the capacity to inhibit either microglial or astrocytic activation, resulting in significant neuroprotection in models of acute CNS lesion, as reviewed previously [44].

In previous studies, we have shown that treatment with the immunosuppressant MMF significantly reduces the extent of neuronal damage [30] and strongly promotes integrity of myelinated long-range projections when applied simultaneously with acute excitotoxic lesion induction [33]. The neuroprotective effects of MMF seemed to be indirectly mediated by counteraction of excessive cytokine secretion and proliferation of glial cells, which are hallmarks of secondary injury cascades [31].

Because clinically suitable drugs need to provide protection even if delivered hours after an incident (when patients can eventually be treated under clinical conditions), we analyzed in the present study the effects of MMF on NMDA-induced temporal patterns of glial proliferation and apoptosis, determined the time frame during which MMF elicits optimum neuroprotection, and investigated the role of MMF on glial scar formation. The well-established experimental model of OHSC was used, which enables analysis of neuron–glia interactions and temporal patterns of excitotoxic neuronal demise without the presence of confounding factors such as inflammatory cells infiltrating from the blood [45-47]. Microglial cells seem to be the only immunocompetent cell type in this model, as blood-borne monocytes and T lymphocytes are apparently absent [48]. Thus, OHSCs are suitable to analyze the effects of MMF on glial cells specifically in the context of neuroprotection.

Knowledge about the temporal patterns of glial proliferation and apoptosis after NMDA lesion is of crucial significance, as the excitotoxicity-mediated neuronal cell decline is generally accompanied by reactive glial cell recruitment and proliferation, and by activation of various apoptosis-related signal transduction pathways that in turn have the potential to exacerbate neuronal injury.

Exposure of OHSCs to NMDA after 6 days *in vitro* induced defined, quantifiable, and reproducible damage to granule cells of the DG, resulting in a pronounced increase in the number of proliferating astrocytes and microglial cells in the early phase after the induction of excitotoxic damage. The proliferation rates of microglial cells were increased to their highest level at 24 hours post-lesion but declined to control levels at 72 hours after injury. Comparably lower proliferation rates were found in astrocytes, although the ratios also increased significantly from 12 to 36 hours post-lesion. These findings indicate that MMF treatment should be started within the first 12 hours after injury at the latest, and should be maintained until 36 hours after injury.

Although excitotoxin-induced neurodegeneration cannot be categorized as one singular mechanism of cell death, but rather involves a combination of necrotic, apoptotic, and autophagic processes [49], key features of apoptosis such as internucleosomal degradation, chromosome fragmentation, and activation of caspases are hallmarks of excitotoxic neuronal death [50,51]. In this regard, we found that in both microglial cells and astrocytes, apoptotic conditions were significantly augmented 24 hours post-lesion and remained increased until 72 hours post-lesion. Notably, quantities of the rate-limiting enzyme IMPDH2 were significantly diminished at 36 and 48 hours after NMDA application, which may be a counter-regulation to exorbitantly increased proliferation rates and might also account for the increased apoptosis indices. These findings emphasize the importance of schedule of administration in the neuroprotective efficacy of MMF.

In fact, delayed but continuous application of MMF on NMDA-lesioned OHSCs resulted in significant neuroprotection only when treatment was implemented at the latest at 12 hours post-lesion. The neuroprotective effects were most pronounced when MMF was applied earlier after the lesion at 4 hours or 8 hours after injury. Neuroprotective effects were paralleled by significant reductions in numbers of IB_4_^+^ microglial cells, thus suggesting that a main source of neuroprotection is a consequence of a potent inhibition of activated, proliferating and neurotoxic glial cells. Previous studies on the time course of excitotoxic neuronal injury after NMDA application showed that numbers of PI^+^ neurons increase rapidly till 24 hours post-lesion and reach a plateau after 36 hours which persists until 72 hours post-lesion [52]. Therefore, late application of MMF will not significantly change the extent of neuronal demise but still be able to effectively suppress glial activation and proliferation.

To figure out the time of efficacy of MMF treatment, we performed a series of experiments with applications of MMF within defined time windows. The crucial time frame in which MMF application displayed the most potent inhibition of glial activation accompanied by significant neuroprotection was in between 12 hours till 36 hours after injury, being in accordance to the above described temporal patterns of neuronal demise. In further investigations on the effects of mycophenolate mofetil, this crucial time frame served as positive control of highest MMF efficacy.

To verify the hypothesis that inhibition of the enzyme IMPDH and subsequent depletion of the purine pool was responsible for the observed effects of MMF, the nucleoside guanosine was applied to refill the purine pools and consequently reverse the MMF-mediated effects. In previous studies, guanosine application was found to potently reverse the inhibitory effects of MMF on the proliferation of LPS-stimulated primary astrocytes and microglial cells [31]. In the current study, guanosine application to unlesioned control OHSCs after 6 days *in vitro* did not impair neuronal viability or numbers of microglial cells. However, guanosine treatment of NMDA-lesioned OHSCs resulted in significant neuroprotection without reductions in microglial cells, indicating direct neuroprotective effects on neurons. In fact, guanosine and guanine nucleotides have emerged as strongly neuroprotective agents that act by directly counteracting glutamate excitotoxicity [53]. Guanine nucleotides are thought to act as glutamate receptor antagonists [54] without affecting the binding capabilities of glutamate or its analogs to glutamate receptors [55,56]. Investigations into the molecular mechanisms underlying the neuroprotective activity indicate that guanosine promotes neuroprotection depending on Ca^2+^-activated K^+^ channels, G-protein-coupled receptors, and modulation of protein kinase (PK)A, PKC, mitogen-activated protein kinase, and phosphatidylinositol-3 kinase pathways in slices subjected to oxygen and glucose deprivation [57-61]. Accordingly, strong innate neuroprotective activity and the various pleiotropic effects of guanosine led to decreased numbers of degenerating neurons that could not further be reduced by MMF treatment. By contrast, application of guanosine to lesioned OHSCs subjected to MMF treatment potently antagonized the strong reductions in the numbers of microglial cells. These findings imply that the MMF-mediated antiproliferative effects depend on depletion of purine metabolites as a consequence of IMPDH inhibition.

The apparent antagonization of MMF effects by substitution of purines should be distinguished from various independent guanosine-induced trophic responses on glial cells. Guanosine has been shown to stimulate proliferation of astrocytes [62,63], and to induce the synthesis and release of neurotrophic and pleiotrophic factors by astrocytes and microglial cells [64,65]. Thus, the various pleiotropic effects described above make guanosine a very sophisticated candidate for the treatment of neuronal injury *in vitro*. However, the strongly hydrophilic nature of this compound will reduce its capability to enter the brain by passing the blood–brain-barrier *in vivo*.

To further investigate the cellular effects of MMF on astrocytes and microglial cells, proliferation and apoptosis assays and scratch-wound model experiments were performed. Treatment of NMDA-lesioned OHSCs with MMF significantly diminished the fraction of Ki-67^+^ proliferating glial cells when treatment was implemented within the crucial 12 to 36-hour neuroprotective time frame, confirming observations of previous studies on LPS-stimulated primary microglial cells and astrocytes [31]. Our present finding of reduced proliferation of microglial cells and astrocytes under the influence of MMF indicates that this substance exerts direct antiproliferative effects on both investigated glial cell types. In addition, MMF has been reported to inhibit the proliferation of various other cell types such as lymphocytes, fibroblasts, mesangial cells, and smooth muscle cells [66-69]. The antiproliferative effect of MMF seems to be based on arrest of the cell cycle in S phase, as was shown in human T cells and monocytes [70]. However, MMF treatment of NMDA-lesioned OHSCs within the crucial 12 to 36-hour time window did not promote or inhibit the initiation of apoptosis.

Similar effects were seen in the *in vitro* scratch-wound model of confluent astrocyte monolayers. The direct mechanical injury is generally accompanied by reactive astroglial scar formation along the lesion site. In confluent monolayers of astrocytes, injuries such as the scratch-induced mechanical break-up of cell–cell connections result in reactive astrocytic activation both in directly wounded cells and in larger cell populations in non-wounded areas throughout the culture. Consecutive signals are thought to spread through intercellular junctions [71] and by release of soluble factors such as cytokines or growth factors [72-74]. Activated astrocytes exhibit characteristic morphological signs of activation such as elongation of hypertrophic processes by realignment and expansion of the GFAP-intermediate filament network or hypertrophy, being both essential for migratory and proliferative responses [75,76].

After the scratch procedure, significant increases were seen in the rate of cell proliferation, predominantly within and around the wound from 12 hours after the scratching procedure until confluence was restored after 48 hours. Experimental groups treated with MMF had a significant decrease in the fraction of Ki-67^+^ cells at 24 and 48 hours after scratching compared with control groups. Furthermore, the scratch-induced gap in these groups was not closed even at 48 hours post- scratching. These findings confirm the inhibitory effects of MMF on astrocyte proliferation, migration, and scar formation.

The modulatory and antiproliferative effects of MMF on microglial cells and astrocytes without direct effects on neurons strengthen the view that this immunosuppressive agent exerts its neuroprotective activity by inhibiting the potentially neurotoxic pro-inflammatory responses that are carried largely by microglia and astrocytes [31]. MMF has not been clinically used in the treatment of acute brain or spinal cord injury in humans to date, but there are reports on neurological benefits of this therapy in various chronic neurological disorders. MMF has been used in the treatment of refractory multiple sclerosis [77,78], multifocal motor neuropathy [79] and chronic inflammatory polyneuropathy [80]. Against the background of strongly beneficial effects on neuronal survival and recovery in numerous *in vitro* studies in combination with promising clinical observations, MMF might be a future candidate for the treatment of acute CNS injuries in humans.

However, despite its broad immunosuppressive abilities making MMF an efficacious drug for transplant and autoimmune diseases, the conceivable risk of infectious complications should be considered. Recipients of solid-organ transplants who receive MMF as part of their post--transplant immunosuppressive regimen seem to have increased susceptibility to infections [81,82] especially cytomegalovirus [83]. However, infectious complications are seen predominantly more than 1 month after initiation of MMF treatment, thus an association with post-transplant renal insufficiency was assumed [84].

In contrast to the long-term applications of MMF, our results indicate that use of MMF as a pharmacological intervention after excitotoxic injury is required only within the first days after injury, thus the risk of post-lesion infections is likely to be low. Nevertheless, *in vivo* experiments are needed to clarify the short-term risks of MMF treatment.

## Conclusions

Taken together, our findings emphasize the therapeutic potential of MMF after acute excitotoxic brain injury. Our results indicate that: 1) delayed administration of MMF until 12 hours after injury potently reduces the extent of secondary neuronal damage; 2) MMF application within the crucial 12 to 36-hour time frame most effectively repealed neuroinflammatory responses and resulted in significant neuroprotection; 3) the neuroprotective agent guanosine antagonized the inhibitory effects of MMF on glial cell activation; 4) MMF effectively suppressed both microglial and astroglial proliferation; 5) apoptosis of glial cells in the aftermath of excitotoxicity was not affected by MMF, and 6) MMF potently counteracted reactive astrogliosis *in vitro*.

These findings emphasize the potential of MMF as a promising and valuable therapeutic candidate in conditions such as cerebral ischemia, spinal cord injury, or trauma.

## Abbreviations

CNS, Central nervous system; DAPI, 4′-6-diamidino-2-phenlyindole; DG, Dentate gyrus; DMEM, Dulbecco’s modified Eagle’s medium; FCS, fetal calf serum; GFAP, Glial fibrillary acidic protein; GCL, granule cell layer; IB4, isolectin B4; IMPDH, Inosine 5-monophosphate dehydrogenase; LPS, Lipopolysaccharide; MEM, Minimal essential medium; MMF, Mycophenolate mofetil; NMDA, N-methyl-D-aspartate; OHSC, Organotypic hippocampal slice culture; PBS, phosphate-buffered saline; PI, Propidium iodide; PK, protein kinase; SDS-PAGE, sodium dodecyl sulfate polyacrylamide gel electrophoresis.

## Competing interests

The authors have no competing interests to disclose.

## Authors’ contributions

FE, CG, PP, and CH performed the experiments. FD designed and coordinated the experiments. FE, MK, IB, and FD analyzed the data and wrote the manuscript. All authors have read and approved the final version of this manuscript.
